# Influenza A H5N1 Clade 2.3.4 Virus with a Different Antiviral Susceptibility Profile Replaced Clade 1 Virus in Humans in Northern Vietnam

**DOI:** 10.1371/journal.pone.0003339

**Published:** 2008-10-06

**Authors:** Mai T. Q. Le, Heiman F. L. Wertheim, Hien D. Nguyen, Walter Taylor, Phuong V. M. Hoang, Cuong D. Vuong, Hang L. K. Nguyen, Ha H. Nguyen, Thai Q. Nguyen, Trung V. Nguyen, Trang D. Van, Bich T. Ngoc, Thinh N. Bui, Binh G. Nguyen, Liem T. Nguyen, San T. Luong, Phuc H. Phan, Hung V. Pham, Tung Nguyen, Annette Fox, Cam V. Nguyen, Ha Q. Do, Martin Crusat, Jeremy Farrar, Hien T. Nguyen, Menno D. de Jong, Peter Horby

**Affiliations:** 1 National Institute of Hygiene and Epidemiology, Hanoi, Vietnam; 2 Oxford University Clinical Research Unit, Hanoi, Vietnam; 3 National Institute for Infectious and Tropical Diseases, Hanoi, Vietnam; 4 Bach Mai Hospital, Hanoi, Vietnam; 5 National Hospital of Pediatrics, Hanoi, Vietnam; 6 National Center for Veterinary Diagnosis, Hanoi, Vietnam; 7 Oxford University Clinical Research Unit, Ho Chi Minh City, Vietnam; 8 Nuffield Department of Clinical Medicine, John Radcliffe Hospital, Oxford, United Kingdom; The University of Hong Kong, China

## Abstract

**Background:**

Prior to 2007, highly pathogenic avian influenza (HPAI) H5N1 viruses isolated from poultry and humans in Vietnam were consistently reported to be clade 1 viruses, susceptible to oseltamivir but resistant to amantadine. Here we describe the re-emergence of human HPAI H5N1 virus infections in Vietnam in 2007 and the characteristics of the isolated viruses.

**Methods and Findings:**

Respiratory specimens from patients suspected to be infected with avian influenza in 2007 were screened by influenza and H5 subtype specific polymerase chain reaction. Isolated H5N1 strains were further characterized by genome sequencing and drug susceptibility testing. Eleven poultry outbreak isolates from 2007 were included in the sequence analysis. Eight patients, all of them from northern Vietnam, were diagnosed with H5N1 in 2007 and five of them died. Phylogenetic analysis of H5N1 viruses isolated from humans and poultry in 2007 showed that clade 2.3.4 H5N1 viruses replaced clade 1 viruses in northern Vietnam. Four human H5N1 strains had eight-fold reduced in-vitro susceptibility to oseltamivir as compared to clade 1 viruses. In two poultry isolates the I117V mutation was found in the neuraminidase gene, which is associated with reduced susceptibility to oseltamivir. No mutations in the M2 gene conferring amantadine resistance were found.

**Conclusion:**

In 2007, H5N1 clade 2.3.4 viruses replaced clade 1 viruses in northern Vietnam and were susceptible to amantadine but showed reduced susceptibility to oseltamivir. Combination antiviral therapy with oseltamivir and amantadine for human cases in Vietnam is recommended.

## Introduction

The host specificity of avian influenza A (H5N1) viruses is generally restricted to birds but occasionally these viruses cross the species barrier to infect mammals, including humans [1]. Since 2003, a total of 373 human infections with highly pathogenic avian influenza (HPAI) virus have been reported to the WHO, of which 236 have been fatal (www.who.int, accessed 24-3-2008). The actual number of cases may be much higher as most infected people live in rural areas with poor access to healthcare and appropriate diagnostic tests.

The genetic plasticity of influenza viruses and previous experience with avian virus-derived human influenza pandemics are the major reasons why the current pandemic of influenza A subtype H5N1 in poultry and wild birds is perceived as such a potential threat to human health. In response to this threat, nations worldwide have based their national contingency plans on the stockpiling of anti-influenza drugs and initiated major efforts to develop H5N1 vaccines. However, the effectiveness of these two strategies will depend on the susceptibility of the pandemic strain, should it emerge, to the stockpiled drugs and the protective immune response elicited by vaccines against the current H5N1 strains. The divergence of influenza H5N1 viruses into several clades challenges these efforts.[2] To date, 10 different genetic clades have been distinguished, some of which have a distinct geographical distribution. The most diverse clade, clade 2, can be subdivided into 5 subclades. Human infections have been caused by (sub)clades 1, 2.1, 2.2, 2.3 and 7 [3].

Between 2003 and 2005, influenza H5N1 outbreaks in poultry and humans in Vietnam were caused by clade 1 viruses [3]–[5]. These viruses were typically resistant to amantadine but susceptible to oseltamivir [3], [6], although the emergence of oseltamivir resistant strains in patients during treatment has been reported [7], [8]. Like most other countries, Vietnam has stockpiled oseltamivir for use in the event of a pandemic. Furthermore, Vietnam has developed a prototype reverse genetics-generated human H5N1 vaccine derived from a clade 1 H5N1 influenza isolate (A/Vietnam/1194/2004).

In 2005 Vietnam started nation-wide poultry vaccination programs which may have contributed to the absence of widespread poultry outbreaks and human infections throughout 2006 [9]. However, in 2007, human infections with HPAI H5N1 viruses began to reappear in northern Vietnam coincident with outbreaks in poultry and waterfowl. The situation regarding poultry outbreaks was described recently, but data on the human cases and their strain characterization are still lacking [5]. Here, we report the clinical characteristics and outcome of patients infected with H5N1 viruses emerging in Vietnam in 2007. H5N1 strains isolated from the human cases were genetically characterized and susceptibility to neuraminidase inhibitors and amantadine were assessed.

## Methods

### Influenza diagnostics

Throat swabs or tracheal aspirates were collected on admission from patients with clinically suspected H5N1 infection in northern Vietnam and sent to the National Institute of Hygiene and Epidemiology (NIHE) for the detection of influenza A H5N1 virus using reverse transcriptase polymerase chain reaction (RT PCR) and viral culture [10]. The national case definition for suspected H5N1 infection is: sudden onset of fever>38°C; difficulty in breathing; infiltrates on the chest X-ray; and a low total leucocyte count in blood. Additional clinical specimens (rectal swabs, blood, pleural fluid), from suspected or confirmed patients that were admitted to the National Hospital of Pediatrics, National Institute of Infectious and Tropical Diseases, and Bach Mai Hospital, all situated in Hanoi, were tested by real time RT PCR and virus isolation at the Oxford University Clinical Research Unit (OUCRU) in Vietnam and at NIHE [11].

For virus isolation, clinical specimens were inoculated onto Madin-Darby Canine Kidney (MDCK) cells in biosafety level 3 laboratories. Virus isolates were identified by RT PCR and hemagglutination inhibition (HAI) with reference antiserum to A/Duck/Hong Kong/820/80 (H5N3). *In vitro* susceptibility of virus isolates to oseltamivir carboxylate was determined by a chemiluminiscent neuraminidase inhibition assay (NA-Star, Applied Biosystems) and to zanamivir by fluorescent neuraminidase inhibition assay, as described previously [12], [13]. A total of 16 virus isolates were tested by neuraminidase inhibition assay: four available human H5N1 strains from 2007, 11 human clade 1 H5N1 viruses isolated in Vietnam between 2004 and 2005, and one human clade 2.3.4 H5N1 strain isolated in Vietnam in 2005. Specimens and strains were stored at −80°C.

Acute and convalescent sera from the first three patients were tested for the presence of H5-specific antibodies by hemagglutination inhibition assay (HAI) using chicken red blood cells and clade 1 (A/Hanoi 3040/2004) and clade 2.3.4 virus (A/Hanoi 31244/2007) antigens. While it is recognised that horse red blood cells are optimal for detecting human antibody responses to H5N1 viruses, horse red blood cells were not readily available in Vietnam.

### Sequencing and phylogenetic analysis

Sequence analysis of the HA gene was performed on six human H5N1 isolates and 11 poultry isolates obtained in 2007. Viral RNA was extracted from virus isolates by use of an RNA extraction kit (QIAamp, Qiagen, Netherlands), according to the manufacturer's protocol. cDNA was synthesized using the A-U12 primer (5′-AGCAAAAGCAGG-3′), which is complementary to the 3′ end of the viral RNA, and reverse transcriptase (SuperScript III; Invitrogen, USA) according to the manufacturer's protocol. The resultant cDNA products were used to amplify the hemagglutinin (HA) gene by a standard PCR method (Proof Start DNA Polymerase; Qiagen, Netherlands) with the following primer pair: H5F: AGC AAA AGC AGG GGT TCA ATC TGT CAA AAT GG and H5R: AGT AGA AAC AAG GGT GTT TTT AAC TAC AAT CTG.

Purified PCR products were sequenced using BigDye Terminator version 3.1 Cycle Sequencing Kit on an ABI 3100 Genetic Analyzer (Applied Biosystems, CA, USA) following the manufacturer's instructions. Haemagglutinin sequences of poultry and human isolates were phylogenetically analyzed together with representative virus sequences available in GenBank, including vaccine candidate strains. Phylogenetic trees of the HA gene segment were created using the maximum likelihood method available in DNASIS-MAX version 02–06 (Hitachi-Japan). Neuraminidase and M2 gene sequencing was performed to assess mutations coding for oseltamivir and amantadine resistance, respectively. The following primers were used for this purpose: NA-534F: TTG CTT GGT CAG CAA GTG C and NA-1149R: TCT GTC CAT CCA TTA GGA TCC for neuraminidase gene and M2F: CTA GTC AGG CCA GGC AAA TG and M2R: ACT GTC GTC AGC ATC CAC AG for M2 gene. Neuraminidase gene sequencing was performed both on human and poultry H5N1 isolates, and M2 sequencing only on human H5N1 isolates.

## Results

**Figure 1:**
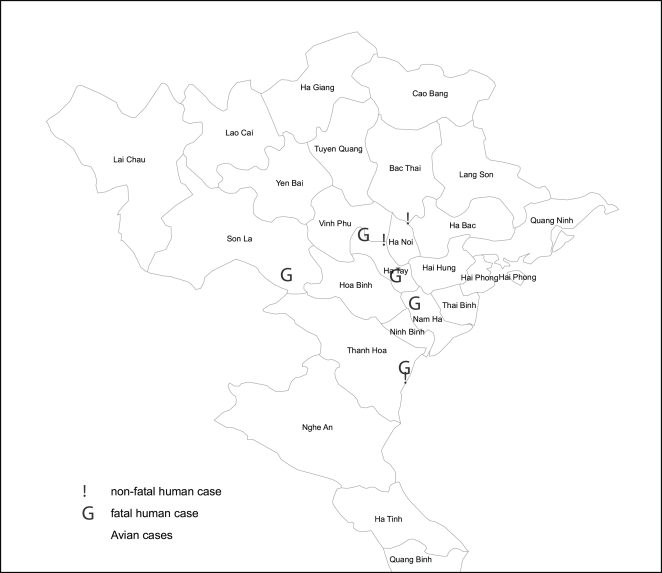
Mapping of poultry and human H5N1 cases in northern Vietnam, 2007.

In 2007, a total of 295 clinical specimens collected from 251 patients in northern Vietnam with suspected H5N1 infection, were tested by RT-PCR at NIHE. Of these, H5N1 infection was confirmed in a total of eight (3.2%) patients: six males and two females (median age 23 y, range: 4–30 y, Table 1). One patient (patient 6) was seven months pregnant. Seven of the patients reported exposure to poultry prior to onset of illness, and five lived in an area with reported poultry outbreaks (Figure 1). Human infections occurred between the start of May 2007 and mid December 2007. The median time: (i) from illness onset to admission was 6 days (range: 4–8), (ii) from admission to discharge or death 5.5 days (range: 1–41), and (iii) from disease onset to death 10 days (range: 7–19). The patients were admitted to four different hospitals in northern Vietnam. No human case was epidemiologically linked with another.

**Table 1:** Clinical features of all eight detected human H5N1 cases in Vietnam, 2007.

	1	2	3	4	5	6ˆ	7	8
**Sex, age (y)**	Male, 29	Male, 19	Male, 30	Male, 20	Female, 28	Female, 26	Male, 15	Male, 4
**Exposure**	chickens sick and died	chickens, ducks, and swans	ducks	raised fighting cock	raised chicken	unknown	dead ducks	chickens sick and died
**Month of admission**	May	May	May	June	June	July	August	December
**Days sick before admission**	5	5	4	6	6	7	6	8
**Days of admission**	27	41	7	2	13	4	1	2
**Time to oseltamivir**	3	3	1	0	1	1	0	1
**Outcome**	survived	survived	survived	died	died	died	died	died
**Fever**	**Y**	**Y**	**Y**	**Y**	**Y**	**Y**	**Y**	**Y**
**Chills**	**Y**	**Y**	NR	NR	NR	**Y**	NR	NR
**Cough**	**Y**	**Y**	**Y**	**Y**	**Y**	**Y**	**Y**	**Y**
**Sore throat**	**Y**	**Y**	NR	NR	N	NR	NR	NR
**Dyspnea**	**Y**	**Y**	**Y**	**Y**	**Y**	**Y**	**Y**	**Y**
**Chest pain**	**Y**	**Y**	**Y**	**Y**	**Y**	**Y**	**Y**	NR
**Vomiting**	**Y**	N	N	N	N	NR	NR	**Y**
**Diarrhoea**	**Y**	**Y**	NR	N	**Y**	N	NR	N
**Temperature (°C)**	40.1	38.2	40	38.7	39	38.2	39.5	38.3
**Pulse (rate/min)**	105	74	100	130	90	105	110	164
**Blood pressure (mmHg)**	100/60	120/60	120/70	110/70	120/80	110/70	90/60	83/38
**Breathing rate (rate/min)**	36	25	23	28	40	60	40	NR
**Lung auscultation**	bilat crepitations	bilat crepitations	bilat crepitations	bilat crepitations	bilat crepitations	bilat crepitations	bilat crepitations	bilat crepitations
**Chest X ray**	bilat infiltrates	bilat infiltrates	bilat infiltrates	bilat infiltrates	bilat infiltrates	bilat infiltrates	bilat infiltrates	bilat infiltrates
**Ventilation**	Y	Y	N	Y	Y	Y	Y	Y
**Pneumothorax**	Y	Y	N	N	Y	Y	unknown	Y
**Pleural effusion**	N	N	N	N	Y	Y	N	Y

ˆ **pregnant.**

**NR: not reported.**

### Clinical features

All patients presented with fever, cough and dyspnea. Mean oxygen saturation was 81% (range: 40–93%) at admission. Pleuritic chest pain was reported by all but one patient in whom pleuritic pain could not be assessed as he was a child (patient 8). Diarrhea was reported in three patients. Conjunctivitis was not a clinical feature. Chest X rays showed bilateral pulmonary infiltration in all patients. Median total leucocyte counts and lymphocyte counts were 4,100 cells/mm3 (range: 2,000–28,700) and 750 cells/mm3 (range: 300–1,700), respectively. The median platelet count was 117,000/mm3 (range: 87,000–164,000). Seven patients required mechanical ventilation early after admission. One patient that survived (patient 3) only received supplemental oxygen and did not require mechanical ventilation. Five patients developed ARDS and pneumothoraces, and in three cases pleural effusion was noted.

### Treatment and outcome

Oseltamivir was administered to all patients within 3 days of admission, corresponding to 5 to 9 days after onset of illness (median: 8 days). After the first two cases had been diagnosed, the time from admission to oseltamivir treatment was reduced from three days to less than one day due to increased awareness. Despite oseltamivir treatment, the mortality was high: 62,5% (5/8). The median time from admission to death was 2 days (range: 1–13). Patient 4 had a very high leucocyte count of 28,700 cells/mm3 and died within 2 days of admission. *Aspergillus fumigatus* was cultured from his sputum. Patient 5 developed ventilator associated pneumonia (VAP) with *Acinetobacter spp* and had undetectable levels of influenza RNA at the time of death, 13 days after admission. All patients received low dose steroids during their admission.

All survivors were admitted in May 2007, but originated from different geographical areas (Figure 1). The fatal cases were all admitted between June and December 2007. Compared to those who died, survivors presented earlier to the hospital after illness onset: median of 5 days versus 6 days (Mann-Whitney U test: P = 0.021) and had better oxygenation parameters at admission: mean breathing rate of 28/min and 42/min (Mann-Whitney U test: P = 0.074).

### Virology

Quantitative influenza RT PCR data were available from four patients: highest viral loads were found in specimens originating from trachea or throat (obtained 6 to 11 days after onset of symptoms), with viral loads ranging from 1.2×10e5 to 1.1×10e6 cDNA copies/mL. Viral RNA could be detected in the plasma (obtained 7 to 9 days after disease onset) of three of five patients tested (mean viral load: 5.2×10e3 cDNA copies/mL). All three patients with influenza virus RNA in plasma died. Detectable H5N1 RNA was also found in the pleural fluid from two patients with effusions both of which were positive with viral influenza RNA in plasma. Drug susceptibility testing of four virus isolates showed mean IC50 values for oseltamivir and zanamivir of 3.1 nM (n = 4) and 1.3 nM (n = 3), respectively (Table 2). Viruses isolated from sequential specimens obtained at admission and three days later on therapy from one patient showed stable susceptibilities to oseltamivir (IC50 4.75 and 3.86 nM respectively). The mean oseltamivir IC50 for eleven clade 1 viruses isolated from Vietnamese patients in 2004 and 2005 is 0.4 nM (range 0.04 to 1.1 nM), illustrating an eight-fold reduced susceptibility of clade 2.3.4 H5N1 viruses as compared to clade 1 viruses. We also tested the oseltamivir IC50 from a clade 2.3.4 strain isolated in 2005 (A/Vietnam/HN 30850/2005, see below), which was found to be 2.0 nM.

**Table 2:** Laboratory data of human H5N1 cases in Vietnam, 2007.

	1	2	3	4	5	6ˆ	7	8
**Sex, age (y)**	Male, 29	Male, 19	Male, 30	Male, 20	Female, 28	Female, 26	Male, 15	Male, 4
**White cell count (/mm3)**	5600	4100	4100	28700	2000	8400	3100	2300
**Neutrophils (/mm3)**	4800	3560	3200	NR	1550	7800	1920	900
**Lymphocytes (/mm3)**	600	460	900	1700	430	300	1180	1300
**Platelets (×10E3/µL)**	87	115	96	132	164	119	108	154
**Na (mmol/L)**	129	148	normal*	131	141	139	142	141
**K (mmol/L)**	2.8	4.5	normal	4.3	4.3	3.1	4.7	3.2
**Cl (mmol/L)**	92	112	normal	95	107	110	ND	110
**Glucose (mmol/L)**	11.3	13	5.5	5.2	15.7	6.8	5.2	7.1
**Creatinine (µmol/L)**	128	104	126	32	125	73	102	42.6
**AST (U/L)**	228	723	82	61	387	199	561	399.9
**ALT (U/L)**	89	684	70	55	113	45	205	63.2
**LDH (U/L)**	ND	2418	ND	2236	3248	ND	ND	ND
**pH**	7.53	7.5	ND	7.46	7.48	7.472	ND	7.35
**PaCO2 (mmHg)**	29.5	31.6	ND	35.6	39.9	26.8	ND	42
**PaO2 (mmHg)**	48.9	61	ND	61	37	38.4	ND	31.1
**HCO3 (mmol/L)**	24.9	24.6	ND	25.2	ND	19.8	ND	20.6
**O2 saturation (%)**	84	88	93	92	91	82	ND	40
**Virus isolated**	Y	NR	NR	Y	Y	Y	Y	Y
**IC50 OST (nM)**	ND	ND	ND	2.7	3.2	2.5	3.8	ND
**IC50 ZNV (nM)**	ND	ND	ND	1.3	1.2	1.3	ND	ND
**Influenza RTPCR** †								
**nose swab/NPA**	NEG	POS	ND	ND	POS	25	ND	ND
**throat/tracheal asp**	3.1×10E5	POS	POS	1.2×10E5	POS	1.1×10E6	POS	POS
**rectum**	NEG	NEG	ND	ND	ND	3.2×10E4	ND	ND
**plasma**	NEG	NEG	ND	NEG	3.1×10E3	7.3×10E3	ND	POS
**pleural fluid**					1.4×10E2	2.7×10E4		
**Serology**								
**Clade 1 antigen**								
**1st serum**	NEG	NEG	NEG	ND	ND	ND	ND	ND
**2nd serum**	NEG	NEG	1∶10	ND	ND	ND	ND	ND
**Clade 2 antigen**								
**1st serum Clade 2.3**	1∶20	1∶10	1∶20	ND	ND	ND	ND	ND
**2nd serum Clade 2.3**	1∶160	1∶160	1∶320	ND	ND	ND	ND	ND

ˆ **pregnant.**

**ND: not done.**

* **within normal range.**

† **if available, quantitative viral loads are presented (cDNA copies/mL).**

**Figure 2:**
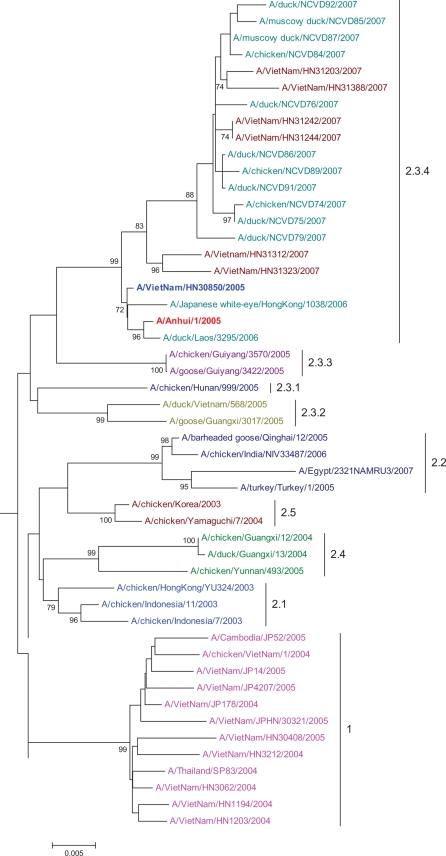
Phylogenetic analysis of hemagglutinin gene sequence of Influenza A H5N1 in humans and poultry in Vietnam 2007 and sequences downloaded from genebank. The numbers to the right of the figure refer to the World Health Organization H5N1 clade designations.

Sequence and phylogenetic analysis of the hemagglutinin gene revealed that the isolated H5N1 viruses were clade 2.3.4 (Figure 2) [14]. Furthermore, we found one of the last human isolates from 2005 to be clade 2.3.4 as well (Figure 2). A polybasic cleavage site motif was present in the HA gene of all sequenced strains. Compared to A/Anhui/1/05 (the current vaccine candidate for clade 2.3), there were 5 to 10 amino acid differences in the HA protein of A/Vietnam/2007 virus (data not shown), which translates to ∼98% homology. In all human H5N1 isolates, no mutations resulting in amino acid substitutions that confer amantadine resistance were found in the M2 gene (L26F, V27A, A30T, S31N, and G34E). In the neuraminidase gene no H274Y or N294S substitution was found. However, in two poultry isolates the I117V substitution was found, conferring reduced susceptibility to oseltamivir.

## Discussion

Here we report on human A H5N1 virus clade 2.3.4 infections in northern Vietnam in 2007. During the same period, clade 1 viruses continued to circulate in poultry in southern Vietnam but did not cause any recognized human infections [5]. The co-circulation of multiple sub lineages of influenza H5N1 in poultry in Vietnam has been previously reported and suggested to have been introduced from southern China where clade 2.3.4 was already dominant [5]. Whilst the clinical presentation and case fatality ratio of the eight patients infected with clade 2.3.4 avian influenza were similar to earlier Vietnam H5N1 cases with clade 1 H5N1 viruses, there were important differences in the antiviral drug susceptibility profile.

The clade 2.3.4 H5N1 strains isolated from four patients showed up to eight-fold reductions in susceptibility to oseltamivir, when compared to human H5N1 clade 1 strains. Additionally, we showed that one clade 2.3.4 H5N1 virus isolated from a human in 2005 already had a reduced susceptibility to oseltamivir. These levels of reduced susceptibility to oseltamivir are of uncertain clinical significance, but may require higher dosing of oseltamivir [15]. All patients in this report were treated with either standard or double dose oseltamivir. It was striking that most patients were brought to medical attention at a rather late stage of their illness which resulted in a delayed initiation of oseltamivir treatment. Better access to diagnostics and treatment in rural areas will help in reducing this delay and will likely improve outcome [3].

Importantly, no amantadine resistance-conferring mutations were observed in the clade 2.3.4 viruses isolated from these patients, in contrast to clade 1 viruses, which are invariably amantadine resistant, and clade 2.1 and 2.2 H5N1 viruses show varying rates of resistance to the adamantanes [3], [6], [16]. Susceptibility to amantadine allows the use of this agent in combination therapy. Oseltamivir treatment in combination with amantadine increases survival in a mouse model and has shown to be well tolerated in healthy volunteers [17], [18]. Combination therapy has as an additional advantage that it may prevent the development of resistance [19]. Therefore, amantadine combined with oseltamivir needs to be considered in patients infected with amantadine sensitive strains, as is also recommended by the WHO [18], [20]. Monotherapy with amantadine should be avoided because of the high risk of resistance development during treatment [21]. For the same reason, the use of amantadine should be prohibited in poultry farming because this will lead to the emergence of amantadine resistant strains in the animal sector with implications for the treatment of human infections [22]. However, the implementation of such an empiric treatment policy in Vietnam will be difficult as amantadine is not registered in Vietnam.

The co-circulation in Vietnam of two distinct highly pathogenic H5N1 strains has important implications for both animal and human health. By reassortment, clade 2.3.4 viruses may arise with the internal gene constellation of clade 1 viruses, as has already been observed in an H5N1 virus isolated from a duck in Vietnam [5]. The emergence of a new clade with different antigenic properties and antiviral susceptibilities than previous outbreak strains in Vietnam in 2007, underscores the need for timely surveillance with further characterization of isolated strains. Though the I117V substitution was present in two poultry isolates, no known mutation was found that could explain the reduced susceptibility to oseltamivir in the four tested human H5N1 strains. This finding emphasizes the importance of phenotypic analysis of drug susceptibility. Since poultry outbreaks precede and outnumber human cases, phenotypic analysis of drug susceptibility should also be routinely conducted on poultry isolates. These data will help the human health care sector with treatment considerations accordingly in a timely fashion.
