# Correction: Influenza A H5N1 Clade 2.3.4 Virus with a Different Antiviral Susceptibility Profile Replaced Clade 1 Virus in Humans in Northern Vietnam

**DOI:** 10.1371/annotation/65ab1e39-3b12-4d9d-90e4-905139530448

**Published:** 2008-10-29

**Authors:** Mai T. Q. Le, Heiman F. L. Wertheim, Hien D. Nguyen, Walter Taylor, Phuong V. M. Hoang, Cuong D. Vuong, Hang L. K. Nguyen, Ha H. Nguyen, Thai Q. Nguyen, Trung V. Nguyen, Trang D. Van, Bich T. Ngoc, Thinh N. Bui, Binh G. Nguyen, Liem T. Nguyen, San T. Luong, Phuc H. Phan, Hung V. Pham, Tung Nguyen, Annette Fox, Cam V. Nguyen, Ha Q. Do, Martin Crusat, Jeremy Farrar, Hien T. Nguyen, Menno D. de Jong, Peter Horby

The symbols in Figure 1 appear incorrectly. Please view the corrected figure here:

**Figure 1:**
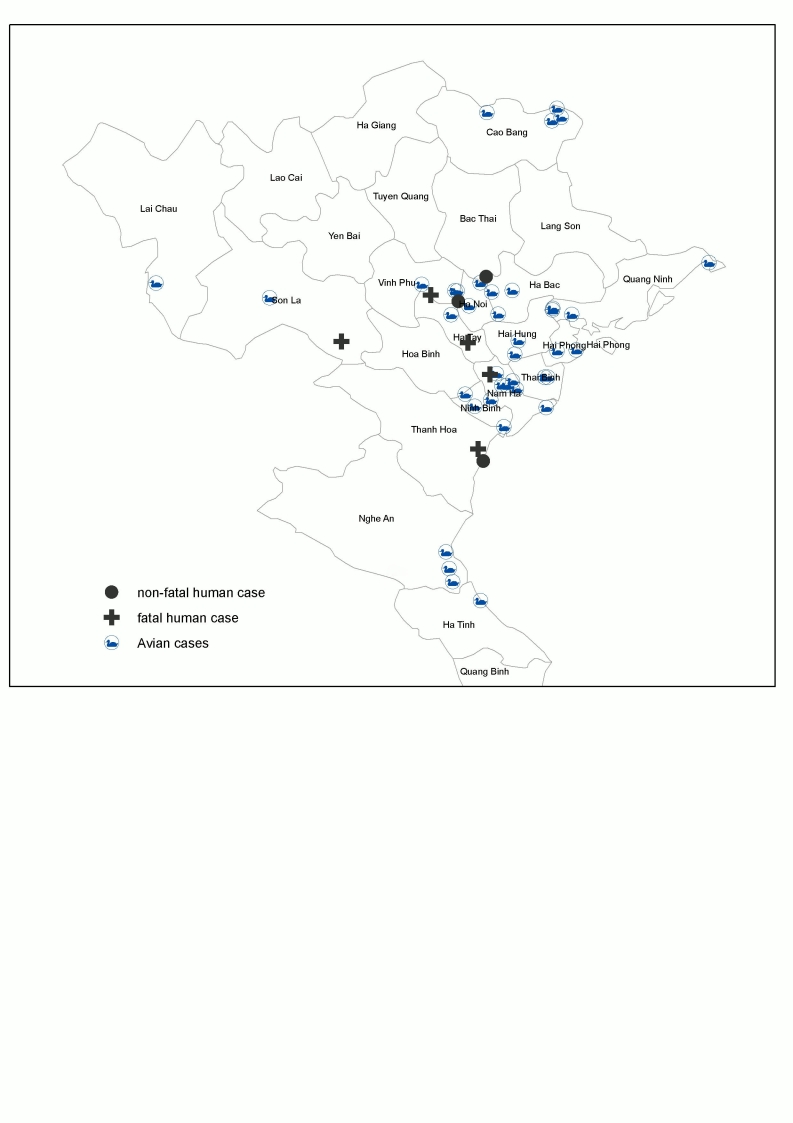
Mapping of poultry and human H5N1 cases in northern Vietnam, 2007.

